# Implementing Clinical Information Systems in Sub-Saharan Africa: Report and Lessons Learned From the MatLook Project in Cameroon

**DOI:** 10.2196/48256

**Published:** 2023-10-18

**Authors:** Georges Bediang

**Affiliations:** 1 Faculty of Medicine and Biomedical Sciences Université de Yaoundé Yaoundé Cameroon

**Keywords:** implementation report, challenges, success factors, Sub-Saharan Africa, Cameroon, healthcare, health care, clinical information, information management, clinical information systems, hospital information systems, data governance

## Abstract

**Background:**

Yaoundé Central Hospital (YCH), located in the capital of Cameroon, is one of the leading referral hospitals in Cameroon. The hospital has several departments, including the Department of Gynecology-Obstetrics (hereinafter referred to as “the Maternity”). This clinical department has faced numerous problems with clinical information management, including the lack of high-quality and reliable clinical information, lack of access to this information, and poor use of this information.

**Objective:**

We aim to improve the management of clinical information generated at the Maternity at YCH and to describe the challenges, success factors, and lessons learned during its implementation and use.

**Methods:**

Based on an open-source hospital information system (HIS), this intervention implemented a clinical information system (CIS) at the Maternity at YCH and was carried out using the HERMES model—the first part aimed to cover outpatient consultations, billing, and cash management of the Maternity. Geneva University Hospitals supported this project, and several outcomes were measured at the end. The following outcomes were assessed: project management, technical and organizational aspects, leadership, change management, user training, and system use.

**Implementation (Results):**

The first part of the project was completed, and the CIS was deployed in the Maternity at YCH. The main technical activities were adapting the open-source HIS to manage outpatient consultations and develop integrated billing and cash management software. In addition to technical aspects, we implemented several other activities. They consisted of the implementation of appropriate project governance or management, improvement of the organizational processes at the Maternity, promotion of the local digital health leadership and performance of change management, and implementation of the training and support of users. Despite barriers encountered during the project, the 6-month evaluation showed that the CIS was effectively used during the first 6 months.

**Conclusions:**

Implementation of the HIS or CIS is feasible in a resource-limited setting such as Cameroon. The CIS was implemented based on good practices at the Maternity at YCH. This project had successes but also many challenges. Beyond project management and technical and financial aspects, the other main problems of implementing health information systems or HISs in Africa lie in digital health leadership, governance, and change management. This digital health leadership, governance, and change management should prioritize data as a tool for improving productivity and managing health institutions, and promote a data culture among health professionals to support a change in mindset and the acquisition of information management skills. Moreover, in countries with a highly centralized political system like ours, a high-level strategic and political anchor for such projects is often necessary to guarantee their success.

## Introduction

Cameroon is a transitional country located in Central Africa. As a resource-limited country, its health system faces many challenges and barriers [1,2]; these include inadequate quality of care, human resources, health infrastructure, poor drug supply, poor health financing, and poor health information management. Yaoundé Central Hospital (YCH) is located in Cameroon’s capital city; it is one of the country's leading national referral hospitals. YCH has several departments, including the Department of Gynecology-Obstetrics, also known as (and hereinafter referred to as) “the Maternity.” The management of clinical information at the Maternity has multiple limitations including those identified by the World Health Organization’s classification of digital health interventions: lack of high-quality and reliable clinical information (issues with data completeness, integrity, and confidentiality), lack of access to the information, and its insufficient use for management and decision-making [3]. This poorly managed clinical information prevents clinicians from accessing information regarding diagnoses, treatments, prescriptions, and previous care [4]. This results in fragmented processes, reduced quality and safety of care, and increased cost of care. Implementing and strengthening robust hospital information systems (HISs) or clinical information systems (CISs) is one of the solutions to these problems [5-8]. The MatLook Project was initiated between the Geneva University Hospitals (HUG) and the YCH to implement an electronic CIS to strengthen the HIS in the Maternity at YCH. This implementation report follows the Guidelines and Checklist for the Reporting on Digital Health Implementations (iCHECK-DH) [9].

## Methods

### Objectives

The general objective of the MatLook Project was to improve the management of clinical information generated at the Maternity at YCH. This implementation report describes the results of the implementation and the associated challenges, success factors, and lessons learned. The primary outcome of this implementation was the completion of the parameterization of MediBoard [10] and services developed, and determination of the level of the deployment of the solution. Secondary outcomes were the development of a procedure manual and determination of the level of its implementation, implementation of training sessions for users, determination of the level of implementation of change management support for users, determination of the level of use of the solution, establishment of project governance or management, development of local leadership to promote the use of the solution, and use of this information for the management of the Maternity or the hospital.

### Technical Design

The objectives of this project were aligned with YCH's strategy, one of its goals being to use ICT to improve YCH's productivity. This project corresponds to categories H and K (electronic medical record and facility management information system, respectively) of World Health Organization’s classification of digital health interventions [3]. At the time this project was implemented, Cameroon did not have a national digital health strategy.

The project was conducted using the HERMES method [11]. This project management method is used in IT, service or product development, and organizational adaptation. It consists of 4 phases: initialization, design, implementation, and deployment. Each step comprises tasks or activities to be performed by roles to achieve the expected results. Each stage leads to a decision or milestone that enables the project to proceed to the next phase. In addition to these phases, plans were developed for risk management, marketing and communication (web portals, newsletters, and information sessions), project quality assurance, and solution testing.

The project was organized into 2 parts. The first part aimed to cover outpatient consultations, billing, and maternity cash management. Billing and cash management were to be handled through the additional development of billing and cash management software that interoperates with this CIS. This first part, including all the 4 phases of the HERMES model, cross-functional project management activities, and users’ training, lasted approximately 15 months. The second part aimed to cover other care processes, such as hospitalization management, delivery management, surgery management, etc.

From a technical point of view, the project consisted in adapting and parameterizing an existing open-source hospital information system called MediBoard [10]. The choice of this solution was based on a comparative study of several HISs with the advantage of being open-source with a dynamic community, the existence of a French version, the fact that several functionalities cover the needs of a hospital located in a resource-limited setting such as Cameroon, and the possibility of making the necessary adaptations relatively quickly [12]. It was also based on the experience of a successful implementation in Mali [6]. MediBoard is an open-source HIS developed in a modular and multilayer web architecture, using Apache, PHP, MySQL, XML, XHTML, JavaScript, CSS, Prototype.js, Smarty, and PEAR. It contains several functionalities such as management of administrative, medical, and nursing patient files; prescription management; consultation appointment management; hospitalization management; billing and accounting management (this service was not adapted to the processes in place, where, for example, people pay before they receive treatment); stock management; human resources management; meal management; quality assurance; incident management; etc.

### Target

The Maternity at YCH was chosen as the pilot unit to implement the solution. It is the second largest maternity unit in Yaoundé after the Yaoundé Gynaecology, Obstetrics and Pediatrics Hospital, which specializes in mother and child care (detailed data not available). This clinical department registers approximately 20,000 patients per year (65% of which are registered through outpatient consultations), 3500 deliveries, and 1000 surgical interventions (63% of which are caesarean deliveries, the remaining 37% of them being ectopic pregnancies, uterine revisions, hysterectomies, myomectomies, cystectomies, etc).

The targets of this intervention were the staff at the Maternity at YCH, including the managers and administrators, health professionals, the team responsible for billing and cash management, and the patients.

### Data

Cameroon has a law (n° 2010/012, December 21, 2010) on cybersecurity and cybercrime, which protects and ensures respect for the privacy of individuals and punishes offences related to the use of information and communication technologies in Cameroon.

Data were collected using computers in consultation rooms, doctors' offices, and treatment rooms. The data were processed and stored at the YCH (data owner) on a data server acquired for this project. The data were disseminated through the intranet network implemented for this project. Several security measures were implemented to ensure data security: physical security (secure rooms dedicated to the server and switches) and computer security and confidentiality (access to the wired intranet network via a dynamic host configuration protocol, implementation of firewalls, management of user profiles, and control of access rights—authentication and authorization—via logins and passwords). Based on their level of confidentiality, the people who had access to these data were reception and triage nurses, doctors, nurses, administrative and financial staff, and patients.

### Interoperability

MediBoard integrates technical interoperability standards such as HL7 (Health Level 7; technical specifications for computerized exchange of clinical, financial, and administrative data between HISs) and HPRIM (harmoniser et promouvoir l'informatique médicale [English: harmonizing and promoting medical informatics] for transmitting information regarding biological examinations in France). It also integrates semantic interoperability standards such as *ICD-10* (*10th revision of the International Classification of Diseases*) and CCAM (Classification Commune des Actes Médicaux [English: Common Classification of Medical Procedures]), which lists all medical procedures to be performed by doctors, midwives, and dental surgeons.

### Participating Entities

At the national level, this project was supported exclusively by the direction of the YCH through the signing of an authorization by the director to carry out a feasibility study and the signing of a decision authorizing the implementation of the project in this hospital. This local institutional support, the needs and requests formulated by the beneficiaries, the potential of the YCH in terms of equipment and human resources, the existence of local expertise in the field of digital health, the individuals in charge of operational management of the project, and the financial guarantees enabled us to implement the project. The project was implemented within the framework of existing cooperation agreements between the YCH, which committed to financing one-third of the budget, and the HUG, which committed to funding two-thirds of the budget. The government was not directly involved because the framework was the existing cooperation agreement between these 2 hospitals.

### Budget Planning

This project was estimated at CHF 60,000 (US $65,144.64): CHF 8000 (US $8685.95) for the adaptation of the MediBoard software; CHF 33,000 (US $35,829.55) for investments (intranet network, servers, computers, switches, etc), communication, and marketing of the project; CHF 14,000 (US $15,200.42) for operations (project management, change management, management of steering, and working meetings with the Maternity user group); CHF 2000 (US $2171.49) for training and support of users; and finally CHF 3000 (US $3257.23) for the evaluation of the solution by an external evaluator of the project. This budget was intended to cover the first and second parts of the project.

### Sustainability

To ensure the sustainability of this project, several actions have been taken, including the alignment of the project’s objectives with those of YCH and its implementation with the agreement of the director of YCH; the commitment of the director to fund one-third of the budget; the establishment of a steering committee including all YCH stakeholders; the integration of the YCH IT manager as the deputy project manager; the creation of a user group to assist the project group in the design and the implementation of the project; the designation of local champions to promote the use of the CIS; and finally, the official launch (supported by a document signed by the director) of this CIS at the Maternity.

### Ethical Considerations

The study does not require ethical approval since it focuses on the implementation of a clinical information system based on an open-source hospital information system. No individual patient data was collected as part of this project.

## Implementation (Results)

### Coverage

The first part of the project covered the Maternity’s outpatient consultations and billing and cash management. The processes covered were the administrative and financial management of patients (administrative data, billing, and payments); management of appointments and clinical information (clinical observations, requests for laboratory and radiological tests, management of the results of these tests, prescriptions for medical consumables, drugs, and medical procedures). The second part did not take place due to nonpayment of one-third of the funding from the YCH.

### Outcomes

The MediBoard software was adapted to manage outpatient consultations. The software parameterization activities included filling in the YCH metadata; adapting forms for entering administrative and sociodemographic data of patients, clinical observations, laboratory and radiology test requests, collection of test result data, medical consumables, and prescription for drugs and medical procedure; defining user profiles (doctor-head of department, gynecologist, resident, reception nurse, student, and secretary) and creating user accounts for maternity health care professionals.

**Figure 1:**
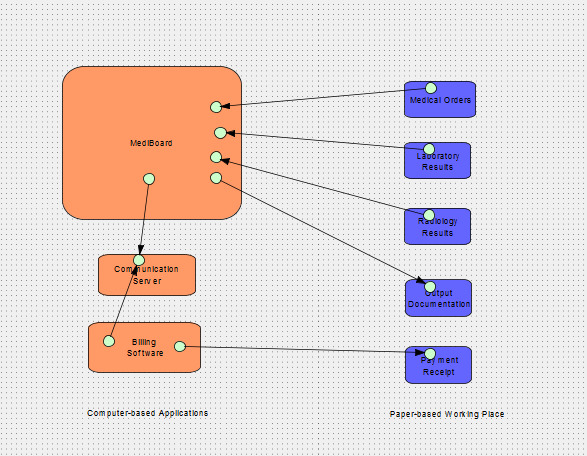
The architecture of the clinical information system of the Department of Gynecology-Obstetrics at Yaoundé Central Hospital (logical level based on the 3LGM^2^ model).

A complementary billing and cash management module, integrating local coding of medical procedures was developed, validated ad hoc, and integrated into the MediBoard HIS (Figure 1) [13]. This software had several functionalities, namely invoice management (creation, modification, and possibility to pay all or part of the invoice); invoice quote management (creation, modification, and transformation of invoice quotes); medical procedure management (editing of medical procedures carried out or the tariffs of these medical procedures); and cash and accounting management (statements of financial entries by the cashier by medical procedure, service, practitioner, period, etc). These services have been deployed at the Maternity at YCH.

An intranet network with a capacity of 40 computers was implemented, and equipment (1 server and 20 laptops) was provided. Security was ensured by physical measures (restriction of access to servers, switches, and consultation rooms) and information and communication technology (ICT) measures (restriction of access to the local network and access to the user account via logins and passwords).

The solution was deployed to cover the Maternity’s outpatient consultations and billing and cash management and was used over 6 months.

To ensure proper functioning of the solution, a user guide and a procedure manual specifying required organizational architecture, processes covered, rules, roles and privileges, and options for entering medical data during the consultation were developed. This procedure manual made it possible, for example, to redefine the workflow for the patient coming for an outpatient consultation.

Users (the staff involved) were regularly trained (before the start of the use of the system and at each staff turnover; this was due to high staff turnover) on the new organizational processes and for proper use of the CIS. This training was provided by a dedicated staff explicitly recruited in the project for this task. In total, 80 people were trained, including senior gynecologists, gynecology residents, medical students, nurses in charge of reception and recording patients’ parameters, and administrative and financial staff. This was a 100% increase on the initial target of people who could benefit from the training during this stage of the project (part 1).

Support staff was recruited to provide specific support to users of the CIS. Computer maintenance was shared between the project team and the YCH IT staff.

An evaluation of the use of this system carried out 6 months after the use of the CIS revealed 113 users, 1278 patient records, and 960 medical consultations recorded, corresponding to a financial income of approximately CHF 8000 (US $8685).

In terms of project governance or management, the project was organized as follows: a steering committee comprising the hospital’s administrative, business, and financial managers to steer the project; a project team comprising external and internal skills in medical informatics, informatics, and project management to implement the project; and a user group comprising health professionals at the Maternity (1 department head physician; 2 gynecologists; 1 anesthesiologist; 4 care coordinators for the outpatient consultations, delivery room, hospitalization, and operating room; 1 nurse; and 1 midwife) to monitor the project activities and to verify that they meet the users’ needs. A marketing and risk management plan for the project has been drawn up. A communication based on the distribution of posters presenting the advantages of the new solution was carried out for the health care professionals and the patients.

Regarding leadership, the 2 heads of department for maternity units A and B were involved as local champions (health professionals of the Maternity who have adopted IT and are ready to provide leadership for their integration in health care in motherhood) to promote the use of the CIS.

Due to the short evaluation period, we could not objectify using the dashboard indicators in the management of the Maternity or the hospital.

### Lessons Learned

#### Success Factors

Several factors contributed positively to the progress of the project—these include the feasibility and implementation agreements of the MatLook project issued by the YCH management; the implementation of a project organization integrating the steering committee, the project group, and the user group; the management of the project based on a robust methodology (HERMES model); the involvement of local champions to ensure leadership and to promote the use of this system; the success of software parameterization and development activities; the realization of several communication and marketing activities for the project and the ongoing training and support of users; and the feasibility and implementation agreements of the MatLook project issued by the YCH management.

#### Challenges

Throughout the project, several challenges or barriers were encountered. These included a change of director of the YCH (3 months after the official launch of the project); lack of leadership from local champions; lack of standard care procedures or protocols; resistance to change; insufficient ICT skills among maternity staff; lack of staff to enter data into the electronic CIS; high staff turnover leading to an increase in the frequency of training sessions on the use of the CIS; poor promotion and valorization of this project within the institution; and an increase in workload due to the diversity of supports to be completed (paper-based service registers coupled with the electronic CIS).

Finally, another challenge was about the reliability and confidentiality of the data collected and the level of responsibility of residents and students for these data. Due to a lack of ICT skills, senior gynecologists gave their logins and passwords to residents or students to document patients’ case files in the electronic CIS on their behalf. This last problem, relating to the exchange of logins and passwords among gynecologists, residents, and students, was identified as soon as the CIS was introduced. To mitigate this, we carried out an awareness-raising campaign among users, demonstrating the risks of this practice and urging them to stop. In addition, we held a meeting with the user group to find more sustainable solutions. The solutions proposed were organizing workshops to strengthen gynecologists' data entry skills; making consultation notebooks with tracers so that stubs could be used for data entry after patients' encounters; and recruiting medical secretaries dedicated to this data entry activity. Unfortunately, none of these measures could be implemented due to a lack of resources.

#### Budget Report

Of the expected CHF 60,000 (US $65,144.64), the project received CHF 40,000 (US $43,360.52; two-thirds of the expected funding) from HUG. These funds were allocated as follows: CHF 8000 (US $8685.95) for the adaptation of the MediBoard software (part 1), CHF 18,500 (US $20,054.24) for the investments (intranet network, computer equipment, and communication and marketing of the project), CHF 9000 (US $9756.12) for operations (project management, change management, management of steering and working meetings, and incentives for health professionals to document cases), CHF 3000 (US $3257.23) for training and support of users, and finally, CHF 1500 (US $1626.02) for the transport of some equipment (servers and computers) from Geneva to Yaoundé and bank account fees. The CHF 20,000 (US $21,680.26; one-third) that was to be paid by the YCH was not paid. This money was to be used as follows: CHF 4000 (US $4336.05) for the adaptation of the MediBoard software (part 2), CHF 4000 (US $4336.05) for the investments (computer equipment, communication, and marketing of the project), CHF 6000 (US $6504.08) for operations (project management, change management, management of steering and working meetings, and incentives for health professionals to document cases), CHF 3000 (US $3252.04) for training and support of users, and finally, CHF 3000 (US $3252.04) for the evaluation of the solution.

#### Unintended Consequences

During this project and after implementing some approaches for change management (setting up a user group, identifying local champions, training users, implementing new processes based on the procedure manual, and setting up user support), we experienced an unintended or unexpected event—staff reluctance to use the system after implementation. The reason was the lack of financial motivation. This situation required the project management team to implement a special documentation gratification (per diem introduced to encourage staff to enter patient information into the system). At the end of this documentation gratification fund (approximately 6 months after the beginning of the use of the CIS), users stopped entering data. In addition, due to the lack of funding from the YCH, part 2 of the project could not be continued, which raises the issue of trust and institutional continuity in sub-Saharan Africa.

## Discussion

Implementing HIS or CIS is feasible in a resource-limited country such as Cameroon. The management of this process is complex and critical to the success of the project.

The implementation of this CIS at the Maternity at YCH was carried out following the defined and approved institutional framework (feasibility and implementation agreements issued by the YCH management, mobilization, and cofunding of the HUG partner) and by relying on a methodology (HERMES phase model) adapted to the implementation of such projects. This project enabled the deployment of a functional CIS at the Maternity at YCH covering (in part 1) outpatient consultations and billing and cash management.

As with any project of this type in our setting, where the implementation of HIS or CIS is still at the embryonic stage, this project registered successes but also many challenges, which have been described in detail in the *Implementation (Results)* section.

Publications describing such implementations in Africa have highlighted similar difficulties: low staff involvement, lack of project management skills, insufficient ICT skills among users, lack of political and administrative support for the project, lack of an interoperability framework, low funding, and lack of logistical support and system maintenance [8,14-18].

In addition, a study evaluating the main factors of failure in implementing HISs in several African hospitals showed that the main difficulties encountered were not technical [8]. They were mainly human, cultural, social, administrative, and environmental, such as skepticism, resistance to change, insufficient ICT skills, poor organization (operating in silos), unrealistic implementation deadlines, insufficient technical support, and lack of support for users after implementation [8,17].

On the other hand, factors strongly associated with success were adequate allocation of resources (infrastructure, human, and financial), good communication, good planning and project management, ability to reorganize the institution to adapt to new processes, local buy-in from stakeholders, implementation based on a holistic approach (integration of all services), highlighting successes to motivate users and reduce mistrust, adequate technical support, perceived usefulness and user satisfaction [6,16,19,20]. To increase the chances of success, in addition to technical aspects (software and equipment) and human resources, good planning, stakeholder commitment, and in-depth change management were required [7]. It is also essential to prioritize design: “enhancing consultations during the intervention design, better consideration of implementation challenges during the design and better recognition of relations between different influences” [21].

By taking into account lessons learned from previous CIS or HIS implementations, including those conducted in resource-limited settings and in addition to technical aspects, we emphasized the management, organizational, leadership, and change management aspects during the project’s implementation. This concerned the representation of all entities (steering committee, project team, and user group), phase-based project management, marketing of the project, management of communication with all stakeholders, drafting and implementation of a procedure manual, use of local champions, change management activities (user awareness–raising activities and allocation of dedicated human resources for responsive users’ support), and finally, the training of users. We also sent a letter and had a meeting with the director of the YCH about the project and raised awareness among health care professionals, managers, and decision makers about the importance of an HIS or CIS in a hospital through scientific conferences and the general public media, etc.

Despite all these approaches, and as shown by our results, beyond the project management, technical, and financial aspects, the other main problems of implementing health or hospital information systems in sub-Saharan Africa lies in digital health leadership, governance, and change management. This digital health leadership, governance, and change management should prioritize data as a tool for improving productivity and managing health care institutions, promote a data culture among health care professionals to support the change of mindset, and the acquisition of information management skills (collection, processing, discussion, and use).

However, the use of good practice in managing a digital health project for setting up a new system by incorporating a good grasp of the project management, technical, organizational, leadership and cultural aspects does not always guarantee its success [18]. In countries with a highly centralized political system such as ours, it is very often necessary for such projects to have a high-level strategic and political anchor (for example, at the level of the ministries responsible for public health or telecommunications or even higher authorities such as the prime minister or the president of the republic). The government has a critical role in developing a vision and creating the foundation upon which innovation activities will be developed [22]. In Mali, for example, the success of the implementation of the HIS at the CHU Mère-Enfant Le Luxembourg was partially based on the support received by the country's first lady. Furthermore, a decision by the minister of health in 2011, with the advent of health insurance in Mali, enabled this tool to be deployed in 72 hospitals nationwide. In the case of our project, at the time of its implementation, Cameroon did not yet have a vision or strategy for digital health. The strategy was adopted in January 2020 and contains 7 strategic objectives covering governance and leadership, legal and regulatory framework, human resources, funding and investment, services and applications, ICT infrastructure, and standards and interoperability [23]. Even if it is still in its embryonic stages, we note that since the adoption of this strategy and following the outbreak of the COVID-19 pandemic, the State of Cameroon is gradually pushing for the adoption and integration of digital health tools into our health system. An operational plan linked to this strategic plan is currently being designed and validated.

## Appendix

Multimedia Appendix 1 Checklist of iCHECK-DH guidelines. iCHECK-DH: Guidelines and Checklist for the Reporting on Digital Health Implementations.

## Abbreviations

CCAM: Classification Commune des Actes Médicaux
CIS: clinical information system
HIS: hospital information system
HL7: Health Level 7
HPRIM: harmoniser et promouvoir l'informatique médicale
HUG: Geneva University Hospitals
*ICD-10*: *10th revision of the International Classification of Diseases*
iCHECK-DH: Guidelines and Checklist for the Reporting on Digital Health Implementations
ICT: information and communication technology
YCH: Yaoundé Central Hospital
